# Genomic and experimental evidence for multiple metabolic functions in the RidA/YjgF/YER057c/UK114 (Rid) protein family

**DOI:** 10.1186/s12864-015-1584-3

**Published:** 2015-05-15

**Authors:** Thomas D Niehaus, Svetlana Gerdes, Kelsey Hodge-Hanson, Aleksey Zhukov, Arthur JL Cooper, Mona ElBadawi-Sidhu, Oliver Fiehn, Diana M Downs, Andrew D Hanson

**Affiliations:** Horticultural Sciences Department, University of Florida, Gainesville, FL 32611 USA; Mathematics and Computer Science Division, Argonne National Laboratory, Argonne, IL 60439 USA; Department of Microbiology, University of Georgia, Athens, GA 30602 USA; Microbiology and Cell Science Department, University of Florida, Gainesville, FL 32611 USA; Department of Biochemistry and Molecular Biology, New York Medical College, Valhalla, NY 10595 USA; Metabolomics Core, UC Davis Genome Center, University of California Davis, Davis, CA 95616 USA

**Keywords:** Metabolite repair, damage pre-emption, reactive enamine/imine, carbamoyl phosphate, FAD-dependent amine oxidase, comparative genomics

## Abstract

**Background:**

It is now recognized that enzymatic or chemical side-reactions can convert normal metabolites to useless or toxic ones and that a suite of enzymes exists to mitigate such metabolite damage. Examples are the reactive imine/enamine intermediates produced by threonine dehydratase, which damage the pyridoxal 5'-phosphate cofactor of various enzymes causing inactivation. This damage is pre-empted by RidA proteins, which hydrolyze the imines before they do harm. RidA proteins belong to the YjgF/YER057c/UK114 family (here renamed the Rid family). Most other members of this diverse and ubiquitous family lack defined functions.

**Results:**

Phylogenetic analysis divided the Rid family into a widely distributed, apparently archetypal RidA subfamily and seven other subfamilies (Rid1 to Rid7) that are largely confined to bacteria and often co-occur in the same organism with RidA and each other. The Rid1 to Rid3 subfamilies, but not the Rid4 to Rid7 subfamilies, have a conserved arginine residue that, in RidA proteins, is essential for imine-hydrolyzing activity. Analysis of the chromosomal context of bacterial RidA genes revealed clustering with genes for threonine dehydratase and other pyridoxal 5'-phosphate-dependent enzymes, which fits with the known RidA imine hydrolase activity. Clustering was also evident between Rid family genes and genes specifying FAD-dependent amine oxidases or enzymes of carbamoyl phosphate metabolism. Biochemical assays showed that *Salmonella enterica* RidA and Rid2, but not Rid7, can hydrolyze imines generated by amino acid oxidase. Genetic tests indicated that carbamoyl phosphate overproduction is toxic to *S. enterica* cells lacking RidA, and metabolomic profiling of Rid knockout strains showed ten-fold accumulation of the carbamoyl phosphate-related metabolite dihydroorotate.

**Conclusions:**

Like the archetypal RidA subfamily, the Rid2, and probably the Rid1 and Rid3 subfamilies, have imine-hydrolyzing activity and can pre-empt damage from imines formed by amine oxidases as well as by pyridoxal 5'-phosphate enzymes. The RidA subfamily has an additional damage pre-emption role in carbamoyl phosphate metabolism that has yet to be biochemically defined. Finally, the Rid4 to Rid7 subfamilies appear not to hydrolyze imines and thus remain mysterious.

**Electronic supplementary material:**

The online version of this article (doi:10.1186/s12864-015-1584-3) contains supplementary material, which is available to authorized users.

## Background

Many metabolites are prone to spontaneous and enzymatic side-reactions that form damaged compounds – a phenomenon known as metabolite damage [1-3]. Damaged metabolites are often unusable and may become toxic if they accumulate, so they must be dealt with [4]. Two common solutions are (i) to repair the damaged metabolite by reconverting it to its original form [5], or (ii) to pre-empt damage by converting a potentially harmful compound into a benign one before damage occurs [5]. Cells may also simply excrete damage products [6,7].

Certain members of the diverse and widely distributed YjgF/YER057c/UK114 family were recently shown to pre-empt metabolite damage by deaminating reactive intermediates of the branched-chain amino acid (BCAA) biosynthesis pathway, and were accordingly named RidA (reactive intermediate deaminase A). Specifically, the isoleucine biosynthesis enzyme threonine dehydratase produces enamines that tautomerize to imines (both of which are very reactive), and RidA proteins catalyze hydrolysis of the imines to 2-oxoacids (which are relatively stable) [8,9]. In bacteria and plants, the serine-derived enamine 2-aminoacrylate (2AA) can attack the pyridoxal 5'-phosphate (PLP) cofactor of branched-chain aminotransferase, which inactivates this enzyme and perturbs BCAA synthesis; RidA proteins forestall this damage by hydrolyzing the corresponding imine 2-iminopropanoate (2IP) [9,10]. 2AA could in principle attack any PLP-enzyme, and has been shown to inactivate alanine racemase [11], serine hydroxymethyltransferase [12], and aspartate aminotransferase [13], so that the protective effects of RidA likely extend beyond BCAA biosynthesis.

A few other disparate activities have been assigned to YjgF/YER057c/UK114 family members. These include *in vitro* molecular chaperone activity for *Drosophila melanogaster* DUK114 [14], endoribonuclease activity for rat L-PSP [15], and specialist chorismatase [16] and 2-aminomuconate deaminase [17,18] activities in bacterial aromatic metabolism.

There is good reason to think that YjgF/YER057c/UK114 family proteins have other – and widespread – roles in metabolic processes. The family occurs in nearly every organism in all domains of life, and some species encode multiple members. *Salmonella enterica*, for instance, has three YjgF/YER057c/UK114 family genes, and *Streptomyces coelicolor* has eleven. Although YjgF/YER057c/UK114 proteins or domains are uniformly small (~130 amino acids), their sequences are diverse and some members share <8% sequence identity [19,20]. The phylogenetic distribution pattern and sequence diversity strongly imply multiple roles, and several have been suggested in pyrimidine degradation [21,22], mitochondrial maintenance [23,24], and metabolic regulation [25].

To explore additional functional roles, we made phylogenetic and comparative genomic analyses of the YjgF/YER057c/UK114 family. This work enabled prediction of two novel roles for RidA proteins, for which experimental support was obtained by biochemical, genetic, and metabolomic approaches.

## Results

### Phylogenetic analysis and nomenclature of the YjgF/YER057c/UK114 family

**Figure 1:**
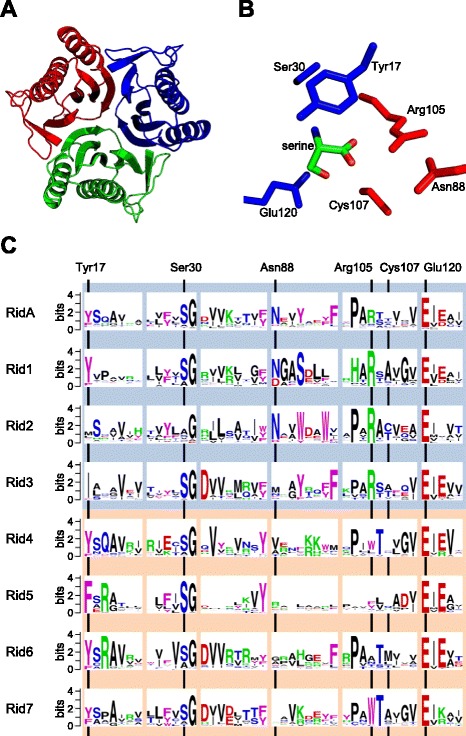
**Sequence features of Rid family members. (A)** Typical trimeric organization of a RidA protein, *Escherichia coli* TdcF. **(B)** TdcF active site with bound serine molecule. Residues of adjacent monomers are colored red or blue. **(C)** Sequence logos show the conservation and relative frequencies of residues in the archetypal RidA and seven subfamilies (Rid1-Rid7). The six regions shown correspond to the footprint regions used to differentiate the subfamilies.

YjgF/YER057c/UK114 family proteins are split into eight subfamilies in the NCBI Conserved Domain Database (see cd00448: YjgF_YER057c_UK114_family), which uses a position-specific scoring matrix to determine conserved domain footprints that imply potential functional sites [26]. Each subfamily could accordingly have distinct functional activities. We henceforth refer to the whole family as the Rid family, to the subfamily containing the characterized RidA proteins as the RidA subfamily, and to the other seven subfamilies as Rid1 through Rid7 (Table 1). This nomenclature meshes with that used in previous studies [8-11]. A subset of RidA proteins (which we term 3x-RidA) has three RidA domains fused in tandem (e.g. EF_0115 in *Enterococcus faecalis* V583). As Rid proteins typically assemble into trimers [19] (Figure 1A) the 3x-RidA proteins are presumably covalently linked trimer units. Other arrangements such as fusions of two or four Rid domains occur, but are rare and so were excluded from this study.

**Table 1:** **Rid subfamily nomenclature**

**subfamily**	**Annotation in SEED database**	**Annotation in NCBI CDD**
RidA	RidA/YER057c/UK114 superfamily protein	YjgF_YER057c_UK114_family
Rid1	RidA/YER057c/UK114 superfamily, group 1	YjgF_YER057c_UK114_like_1
Rid2	RidA/YER057c/UK114 superfamily, group 2, YoaB-like protein	YjgF_YER057c_UK114_like_2
Rid3	RidA/YER057c/UK114 superfamily, group 3	YjgF_YER057c_UK114_like_3
Rid4	RidA/YER057c/UK114 superfamily, group 4	YjgF_YER057c_UK114_like_4
Rid5	RidA/YER057c/UK114 superfamily, group 5	YjgF_YER057c_UK114_like_5
Rid6	RidA/YER057c/UK114 superfamily, group 6	YjgF_YER057c_UK114_like_6
Rid7	RidA/YER057c/UK114 superfamily, group 7, YjgH-like protein	YjgH_like

Annotation of Rid subfamilies in the SEED and NCBI databases.

Crystal structures and mutagenesis studies have identified functionally important residues in the RidA subfamily. The highly conserved Arg107 forms salt bridges with the carboxylate oxygens of benzoate in human hp14.5 [27] and the corresponding Arg105 hydrogen bonds with the carboxylate oxygens of serine, threonine, and 2-oxobutanoate in *Escherichia coli* TdcF (paralogous to *S. enterica* RidA) [28] (Figure 1B). Changing this arginine to alanine almost completely abolished imine-hydrolyzing activity in plant and *S. enterica* RidA proteins [8,9]. It has been suggested that conserved Tyr17 and Glu120 residues of *E. coli* TdcF also play a role in substrate binding and positioning of a water molecule used for imine hydrolysis, but replacing the corresponding residues with alanine had little effect on the activity of *S. enterica* RidA [8].

We analyzed residue conservation for all the Rid family proteins listed in the NCBI Conserved Domain Database, paying particular attention to the predicted active site residues (Figure 1C). The Rid1, Rid2, and Rid3 subfamilies retain the conserved arginine and glutamate residues found in RidA. The Rid4, Rid5, Rid6, and Rid7 subfamilies have the glutamate residue but the arginine residue is replaced by tryptophan in Rid4 and Rid7, and is variable in Rid5 and Rid6. The tyrosine is conserved, or conservatively replaced by phenylalanine, in all subfamilies except Rid3 where it is replaced by isoleucine and Rid2 where it is variable. The presence or absence of the critical arginine residue thus separates Rid subfamilies into two groups: (i) subfamilies RidA and Rid1-Rid3 that have this residue and are predicted to hydrolyze imines, and (ii) subfamilies Rid4-Rid7 that lack it and presumably serve other functions. Finally, it should be noted that a small minority (~10%) of RidA proteins have a serine residue instead of arginine, suggesting there may be functional diversity even within the RidA subfamily (Figure 1C).

**Figure 2:**
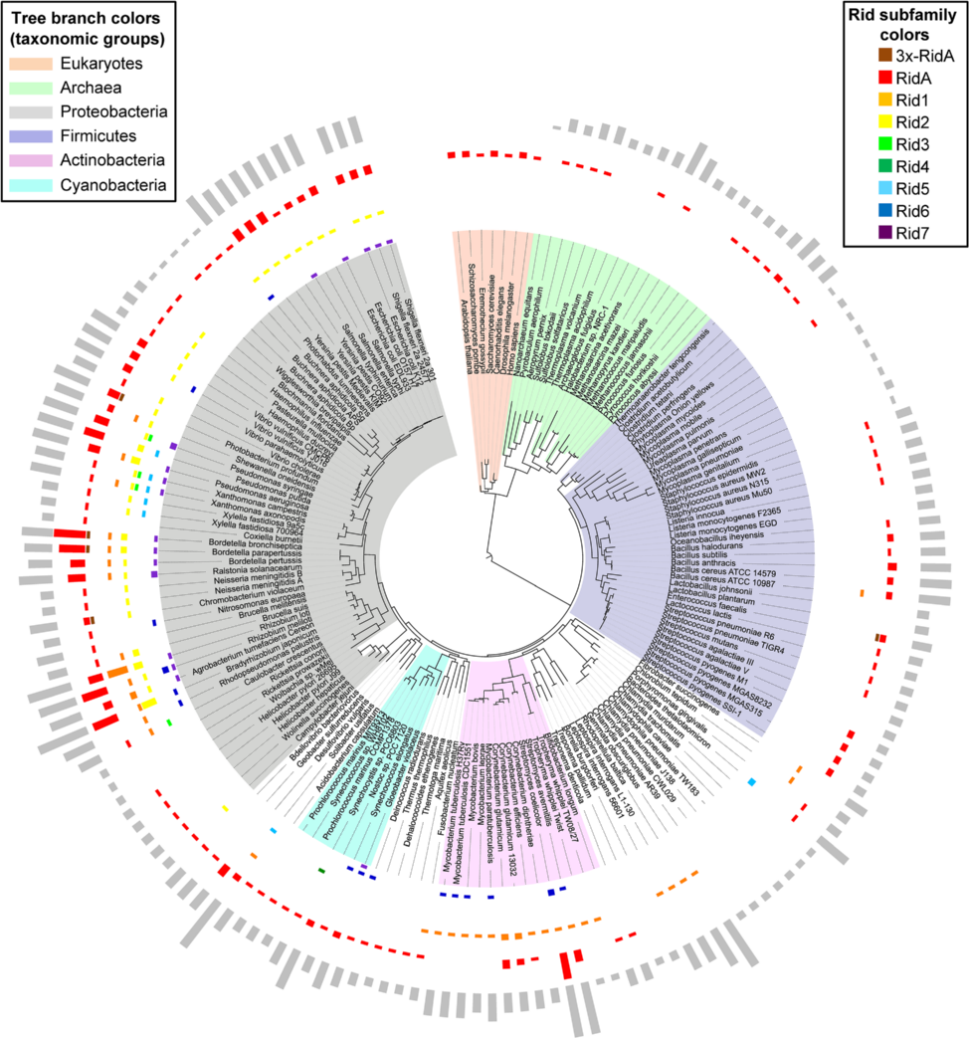
**Phylogenetic distribution of the Rid family.** Phylogenetic distribution of the Rid family. Occurrence of Rid family members is mapped onto a Tree of life (http://itol.embl.de). This phylogenetic tree of all three domains of life was constructed by Ciccarelli et al., 2006 [29] using the alignment of a concatenation of 31 orthologous proteins occurring in ~200 representative species with sequenced genomes. Bar size indicates the number of RidA gene copies per subfamily present in each of the genomes shown. Relative genome size is indicated by the size of the outermost gray bars (excluding eukaryotes).

To assess their phylogenetic distribution, we mapped Rid family members onto a phylogenetic tree containing almost 200 sequenced organisms representing all three domains of life [29] (Figure 2). This mapping showed that the RidA subfamily is widely distributed among all domains of life whereas the other seven subfamilies occur primarily in bacteria, especially proteobacteria. This pattern suggests that the RidA subfamily is archetypal and gave rise to the other subfamilies. We also mapped genome size onto the phylogenetic tree and found that it correlated positively (*r* = 0.69) with the number of Rid genes per genome (Additional file 1). Similar correlations have been observed for other large gene families [30].

### Comparative genomic analysis of the Rid family

We used comparative genomic analysis to explore the possibility that Rid family proteins have functions besides hydrolysis of threonine dehydratase-derived imines, as suggested by their sequence diversity and phylogenetic distribution. The analysis was made on a representative set of 981 prokaryotic genomes (Additional file 1) (see Methods for details) using the SEED database and its tools, which enable ready detection of gene clustering patterns [31]. Gene clustering in prokaryotes strongly implies a functional relationship, particularly when clusters occur in more than one configuration and in diverse organisms [32]. Full results of the analysis are available at the SEED database (see Methods).

**Figure 3:**
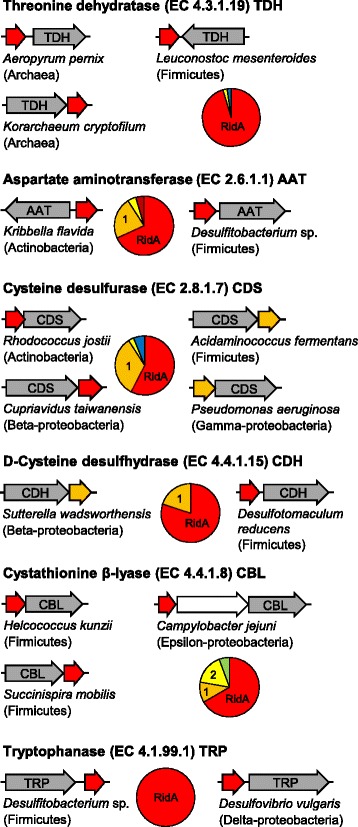
**Rid family member genes cluster on prokaryotic chromosomes with genes encoding various PLP-dependent enzymes.** Gene models show the orientation of clustered genes in representative prokaryotic genomes. Pie charts show the relative frequency with which clustering occurs with various Rid subfamilies. The color scheme for Rid subfamilies is the same as in Figure 2. Genes in white have unrelated functions.

Because RidA can pre-empt damage to various PLP-dependent enzymes by hydrolyzing the reaction products of threonine dehydratase, we expected clustering between the corresponding genes. Indeed, RidA genes were found to cluster consistently with threonine dehydratase genes in bacteria and archaea (Figure 3). Aspartate aminotransferase, which is prone to damage by 2AA (13), also clusters with Rid genes in diverse bacteria – usually with RidA and less often with Rid1 (Figure 3). L-Cysteine desulfurase and D-cysteine desulfhydrase likewise cluster in diverse bacteria with RidA, or sometimes Rid1 (Figure 3). Like threonine dehydratase, these enzymes can form the damaging intermediate 2AA [33,34]. RidA, Rid1, or Rid2 genes also cluster with the PLP enzymes cystathionine β-lyase and tryptophanase, both of which can produce 2AA [35,36] (Figure 3). The above instances of clustering, while expected, clearly confirm that gene clustering patterns can reflect Rid gene function.

**Figure 4:**
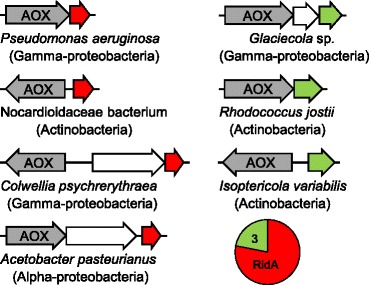
**Rid family member genes cluster on bacterial chromosomes with genes belonging to the amine oxidases (AOX) family.** The layout and color scheme are as in Figure 3.

**Figure 5:**
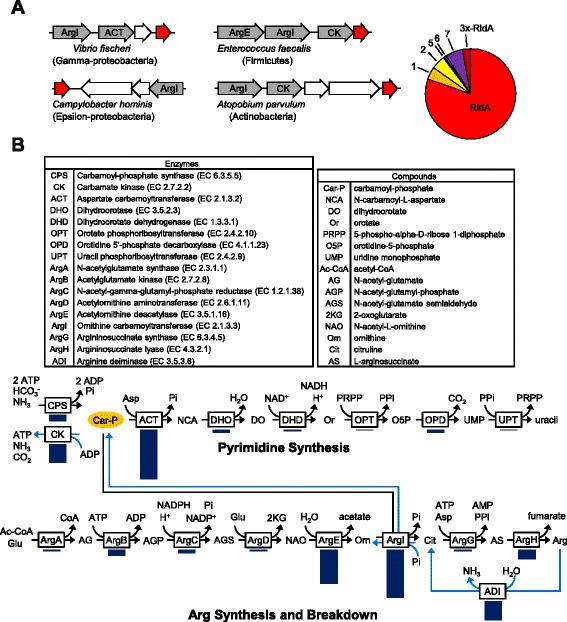
**Rid family genes cluster on bacterial chromosomes with pyrimidine and arginine metabolism genes, particularly those related to carbamoyl phosphate. (A)** The layout is the same as in Figure 3. **(B)** The metabolic pathways of pyrimidine and arginine synthesis (black arrows) and breakdown (light blue arrows) are shown. Dark blue bars under each enzyme indicate the relative proportion of genomes (in the set of 981 genomes analyzed) in which each gene of pyrimidine or arginine metabolism is clustered on the chromosome with a Rid family gene. The longest bar (ACT in the pyrimidine pathway) corresponds to 58 instances of clustering.

We were therefore intrigued to find two additional clustering patterns in bacteria. First, RidA or sometimes Rid3 genes frequently cluster with FAD-dependent amine oxidase family genes (Figure 4). As FAD-dependent amine oxidases produce an imine intermediate that spontaneously hydrolyzes to a 2-oxoacid [37,38] it is possible that RidA and Rid3 accelerate the hydrolysis of these imines, as RidA does for imines produced by threonine dehydratase. Second, RidA and occasionally other Rid genes cluster with genes of arginine or pyrimidine metabolism (Figure 5A). About 80% of clustering occurred with RidA genes; Rid7 genes were the next most common but >70% of these occurred in a cluster that also contained RidA.

To home in on functional associations, we mapped the frequency with which Rid genes cluster with individual arginine and pyrimidine metabolism genes. This analysis showed that clustering centered on genes for carbamoyl phosphate-related enzymes, none of which have a PLP cofactor or are known to form enamines or imines (Figure 5B). This association suggests a novel role for Rid proteins in carbamoyl phosphate metabolism.

### RidA hydrolyzes reactive imines produced by amino acid oxidases

To assess the ability of Rid family proteins to hydrolyze imines produced by FAD-dependent amine oxidases we used an assay based on the ability of imines to react rapidly with semicarbazide to produce semicarbazones, which absorb in the UV range [9,39]. The competition between semicarbazone formation and Rid-mediated hydrolysis of the enzymatically produced imines can thus be monitored spectrophotometrically. We chose to assay the three Rid proteins of *S. enterica* because *S. enterica* RidA is well studied and so can serve as a benchmark for imine-hydrolyzing activity, and because *S. enterica* Rid2 (YoaB) has the catalytically important arginine residue and *S. enterica* Rid7 (STM1549) does not.

**Figure 6:**
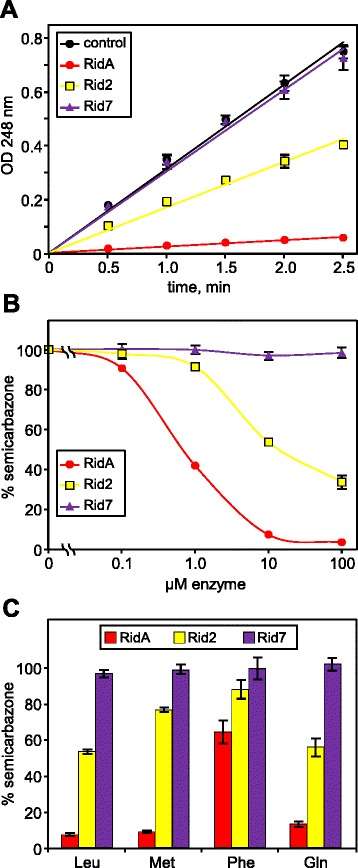
***S. enterica*** **RidA and Rid2, but not Rid7, accelerate hydrolysis of imine products of L-amino acid oxidase. (A)** Semicarbazone formation in assays containing leucine with or without 10 μM Rid protein. **(B)** Semicarbazone formation in assays containing leucine and various amounts of Rid protein. **(C)** Semicarbazone formation in assays containing various amino acid substrates and 10 μM Rid protein. Error bars indicate SE from at least three replicate assays. Data in **B** and **C** represent the amount of semicarbazone formation as a percent of control assays containing no Rid protein.

Purified L-amino acid oxidase (LOX) from *Crotalus adamanteus* venom was used as a model FAD-dependent amine oxidase because it has been used previously in assays containing semicarbazide [39]. Recombinant *S. enterica* RidA, Rid2, and Rid7 were purified to near-homogeneity by Ni^2+^-affinity chromatography (Additional file 2: Figure S1). Rapid semicarbazone formation was observed when LOX was incubated with leucine and semicarbazide (Figure 6A). Addition of 10 μM RidA, Rid2, or Rid7 caused a 93%, 46%, or <3% decrease in the rate of semicarbazone formation, respectively (Figure 6A, B). To better assess the ability of the three *S. enterica* proteins to hydrolyze the leucine-derived imine, we varied the concentrations of the Rid proteins included in the assay. RidA was the most active, reducing the rate of semicarbazone formation by 50% at less than 1 μM compared to about 10 μM for Rid2 (Figure 6B). Rid7 did not significantly reduce semicarbazone formation even at 100 μM, showing that it cannot hydrolyze the leucine-derived imine (Figure 6B).

We then tested the ability of the *S. enterica* proteins to hydrolyze other amino acid-derived imines. First, the rate of semicarbazone formation was determined in assays containing LOX and various amino acid substrates without Rid proteins (Additional file 2: Table S1). Amino acids with polar and charged side groups proved to be very poor substrates, and thus non-charged methionine, phenylalanine, and glutamine were chosen to test further. The amount of LOX in the assay was adjusted so that the rate of semicarbazone formation was the same for each amino acid substrate, thus allowing direct comparison of imine-hydrolyzing capabilities. At 10 μM, Rid7 did not significantly reduce semicarbazone formation for any amino acid tested, indicating that it cannot hydrolyze any of the amino acid-derived imines (Figure 6C). RidA hydrolyzed the methionine-derived imine nearly as effectively as the leucine-derived imine, reducing the rate of semicarbazone formation by 91% compared to 93%, and was only slightly less effective (86% reduction in rate of semicarbazone formation) in hydrolyzing the glutamine-derived imine (Figure 6C). Rid2 hydrolyzed the glutamine- and leucine-derived imines at similar rates (44% and 46% reduction in semicarbazone), and hydrolysed the methionine-derived imine more slowly (23% reduction in semicarbazone). RidA and Rid2 also hydrolyzed the phenylalanine-derived imine, although relatively slowly, reducing semicarbazone formation by 35% and 12%, respectively. These results show that RidA and to a lesser extent Rid2, but not Rid7, can hydrolyze several imino acids and that RidA and Rid2 differ in substrate preference.

### Genetic evidence supports a RidA-carbamoyl phosphate connection

To explore the functional connection between Rid proteins and carbamoyl phosphate predicted by comparative genomics, we engineered *S. enterica* to accumulate carbamoyl phosphate, and ablated genes encoding each of the three Rid family proteins. *S. enterica* is particularly appropriate for this experiment because its RidA gene clusters with the arginine metabolic genes encoding ornithine carbamoyltransferase and Arg deiminase, the regulatory gene *argR*, the pyrimidine metabolic gene encoding Asp carbamoyltransferase, and carbamate kinase, suggesting that *S. enterica* RidA plays a role in carbamoyl phosphate metabolism. The arginine biosynthesis pathway was blocked by disrupting the gene encoding ornithine carbamoyltransferase (ArgI), and an expression plasmid containing the gene encoding the CarB subunit of carbamoyl phosphate synthetase (CPS) was introduced. This subunit is sufficient to produce carbamoyl phosphate provided that the medium contains ammonium [40]. The plasmid used (pCA24N-*carB*) was isopropyl β-D-thiogalactopyranoside (IPTG)-inducible [41]. We reasoned that an IPTG-inducible growth defect in strains lacking a Rid family member would confirm a role for this Rid protein in carbamoyl phosphate metabolism.

**Figure 7:**
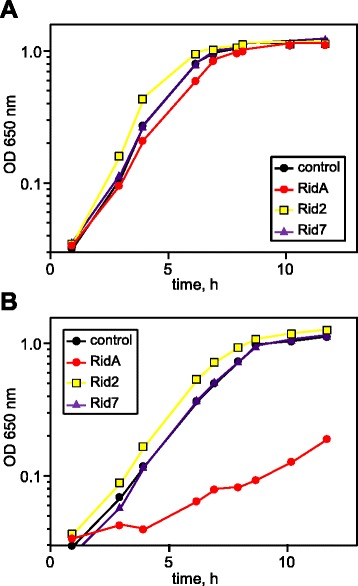
***S. enterica*** **cells lacking** ***ridA*** **are sensitive to induction of carbamoyl phosphate synthetase.** Cells were grown at 37°C in nutrient broth with **(A)** no additions or **(B)** supplemented with 0.1 mM IPTG. Growth was monitored by optical density at 650 nm. All strains contain an insertion in *argI*, the gene encoding ornithine carbamoyltransferase, and harbor plasmid-encoded CarB under the control of an IPTG-inducible promoter. Strains are represented in the figure legends by the Rid protein they lack. Strain names and relevant genotypes are RidA (DM14200), Rid2 (DM14307), Rid7 (DM14223), and control (DM14203). Data shown are representative of at least three independent experiments done in biological triplicate on separate days.

We compared the growth of the control strain containing all three native *rid* genes with strains lacking *ridA*, *Rid2*, or *Rid7*. All strains grew similarly in nutrient broth at 37°C in the absence of IPTG (Figure 7A). When IPTG was included, growth of the control strain, and strains lacking *Rid2* or *Rid7*, was indistinguishable from that in medium without IPTG but the strain lacking *ridA* had a substantial growth defect (Figure 7B). These results indicate that accumulation of carbamoyl phosphate is detrimental to *S. enterica* cells lacking a functional RidA protein, and thus point to a role for RidA in controlling damaged caused directly or indirectly by carbamoyl phosphate.

### Metabolomic analysis indicates division of labor among *S. enterica* Rid proteins

**Figure 8:**
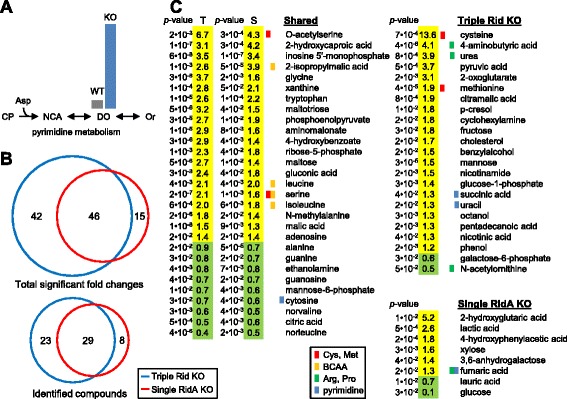
**Metabolomic analysis of** ***S. enterica*** **Rid knockouts reveals widespread metabolic disturbances.** Wild-type *S. enterica* and triple Rid KO (*ridA Rid2 Rid7*; DM14100), and for GC-MS also single RidA KO (*ridA*; DM3480), cultures were grown, harvested, and analyzed as described in Methods. **(A)** HILIC-TOF-MS identified dihydoorotate as having a significant 10.5-fold change in the triple KO. Part of the pyrimidine metabolic pathway is shown (see Figure 5 for abbreviations) with bars indicating the relative amount of dihydroorotate in each sample. **(B)** Venn diagrams summarize the significant (*P* < 0.05; t test) -fold changes (KO/wild-type) for GC-TOF-MS identified and unknown peaks. **(C)** GC-TOF-MS identified compounds with significant -fold changes found in one or both knockouts are listed in order of -fold change (for shared compounds, triple (T) or single (S) Rid KO is indicated) and colored yellow or green to indicate increased or decreased levels in the knockout, respectively. *p*-values are shown to the left of -fold changes. Colored bars adjacent to compound names mark intermediates of metabolic pathways shown in the legend. Data represent six (A, LC-MS) or twelve (**B** and **C**, GC-MS) independent cultures for each treatment.

Two different analytical techniques, LC-MS and GC-MS, were used to explore the metabolic consequences deleting Rid family genes. Wild type and triple (*ridA Rid2 Rid7*) knockout *S. enterica* cells were first profiled using a hydrophilic interaction liquid chromatography-time-of-flight mass spectrometry (HILIC-TOF-MS; i.e. LC-MS) platform to favor detection of highly polar compounds. Samples were collected as they entered late log phase; this harvesting point was selected to maximize the difference between wild-type and triple knockout cells (Additional file 2: Figure S2). Only two metabolites showed significant (*P* ≤ 0.05) accumulations of ≥1.5-fold in knockout cells. Of these, the larger accumulation (10.5-fold) was of dihydroorotate, a pyrimidine synthesis intermediate located one step downstream of the carbamoyl phosphate-dependent step in the pathway (Figure 8A). The smaller accumulation (2.8-fold) was of 4-aminobutyrate, a general stress metabolite. The specificity and size of the dihydroorotate pool size change suggest a role for the Rid family in pyrimidine synthesis, possibly in countering an adverse effect of carbamoyl phosphate (or its breakdown product isocyanate) on dihydroorotate dehydrogenase.

The more general metabolic impact of ablating Rid family members was surveyed using a gas chromatography-time-of-flight mass spectrometry (GC-TOF-MS) metabolomics platform. We compared single *ridA* and triple *ridA Rid2 Rid7* knockout strains with wild-type *S. enterica*. In total, 277 compounds were detected in all three strains, of which 144 were positively identified. We then calculated the relative -fold change for each compound between the mutant strains and wild-type. About 22% of compounds in the single RidA knockout had significant (*P* ≤ 0.05) -fold changes (60 of 277 total compounds; 37 of 143 identified compounds), while ~31% of compounds in the triple knockout showed significant -fold changes (87 of 277 total compounds; 52 of 143 identified compounds) (Figure 8B). Besides confirming that Rid2 and Rid7 cannot replace RidA, these data suggest that Rid2 and Rid7 have roles of their own, i.e. that there is division of labor among *S. enterica* Rid proteins. There was much overlap in the profiles of the two knockout strains; 78% of the compounds that changed significantly in the single knockout also did so in the triple knockout (Figure 8B). Furthermore, the changes in overlapping compounds were always in the same direction (increase or decrease), and were generally of similar magnitude (Figure 8C).

The compounds showing significant -fold changes came from diverse sectors of metabolism. One sector was sulfur metabolism; cysteine showed the largest change of any identified compound – almost 14-fold in the triple knockout (Figure 8C). A second sector was BCAA metabolism; isoleucine and leucine changed in both knockouts, as did other BCAA pathway metabolites (Figure 8C). Several compounds involved in arginine, proline, and pyrimidine metabolism changed, possibly due to a lesion in carbamoyl phosphate metabolism (Figure 8C). Purine-related metabolites were also affected, as were various sugars and sugar derivatives (Figure 8C).

## Discussion

Separating the Rid family into subfamilies, and analyzing patterns of residue conservation and chromosomal clustering in these subfamilies, led to one finding that was broadly predictable from previous work, and two that were not. The broadly predictable finding was that the largest and most widely distributed subfamily, RidA, most probably pre-empts damage by hydrolyzing 2IP (the tautomer of 2AA) that comes from other PLP-dependent enzymes besides the known source threonine dehydratase. 2AA is formed in the normal catalytic or side-reactions of L-cysteine desulfurase [33] and D-cysteine desulfhydrase [34], cystathionine beta-lyase [35], and tryptophanase [36], with each of which RidA clusters (Figure 3).

The first novel finding was that the RidA subfamily, the Rid2 subfamily, and probably the Rid1 and Rid3 subfamilies, which all retain the arginine residue shown to be essential for 2IP hydrolysis [8,9], can hydrolyze imines produced by FAD-dependent amine oxidases. This finding extends the known imine-hydrolyzing function of Rid proteins to a wider range of imines that are formed by enzymes in pathways very different to those involving the imine-forming PLP enzymes. The imine compounds formed by amine oxidases could potentially react with, and hence damage, various cellular components [39,42]. Thus, the physiological implications are that enamine/imine damage to metabolism is not restricted to that from 2AA/2IP and that, collectively, Rid family proteins with the conserved arginine residue could act as wide-spectrum enamine/imine damage-pre-emption enzymes.

The second novel finding was that the RidA subfamily, and possibly other Rid subfamilies, are closely connected to carbamoyl phosphate metabolism. Under physiological conditions, carbamoyl phosphate breaks down spontaneously and rapidly (half-life ~5 min at 37°C) to phosphate and isocyanate, which tautomerizes to cyanate [43]. Isocyanate is a potent damage agent because it reacts readily with amino, thiol, carboxyl, and other groups, and so carbamoylates amino acids, proteins and other molecules [44]. Because isocyanate and carbamoyl groups have some similarity to imines, and RidA proteins have imine hydrolase activity, it is reasonable to infer that this subfamily can pre-empt carbamoylation damage in an analogous way to its action in pre-empting imine damage. In this connection, it may be noted that a simple possibility – that RidA proteins hydrolyze carbamoyl phosphate or isocyanate to release ammonia – was not supported by pilot experiments with *S. enterica* RidA (Additional file 2: Figure S3). Another possibility is that Rid proteins protect a carbamoyl phosphate-related enzyme or metabolite from damage caused by 2AA or another enamine/imine-containing compound.

## Conclusions

The Rid protein family comprises a large, widely distributed – and probably archetypal – subfamily (RidA) and seven smaller subfamilies (Rid1 through Rid7) found mainly in bacteria. The RidA through Rid3 subfamilies share a catalytically critical arginine residue and apparently serve to hydrolyze the reactive imines generated by PLP-dependent enzymes or FAD-dependent amine oxidases, thereby pre-empting the damage these imines would otherwise cause. The RidA subfamily most probably has an additional damage-pre-emption role in carbamoyl phosphate metabolism that has yet to be biochemically defined.

The biochemical activities and physiological functions of the Rid4 through Rid7 subfamilies remain completely unknown. Thus far it is clear that these four subfamilies are most likely not imine hydrolases since they lack the arginine residue on which imine hydrolase activity depends, and because no imine hydrolase activity was detected for *S. enterica* Rid7. And although the Rid1 through Rid3 subfamilies are predicted – and in the case of *S. enterica* Rid2, demonstrated – to have imine hydrolase activity *in vitro*, nothing is known about the *in vivo* substrates for these three subfamilies.

In sum, the Rid family as a whole still has only a few known functions, and additional functions surely remain to be discovered. It is nonetheless clear that RidA proteins are iconic examples of the emerging principle of metabolite damage pre-emption [5]; the same seems likely to prove true of proteins from other Rid subfamilies.

## Methods

### Bioinformatics

Sequences were from GenBank or the SEED database [31]. Sequences were analyzed with the NCBI CDD database and CDTree tool. To analyze residue conservation, protein alignments were taken from NCBI CDD, trimmed with Galaxy tools (https://usegalaxy.org/root), and used to create graphics with WebLogo (http://weblogo.berkeley.edu/) [45,46]. Crystal structure analysis and graphics were made with PyMol (http://www.pymol.org/). Comparative genomic analyses were made with SEED [31]. Figure 2 was created using iTOL tools [47,48].

A set of 981 representative genomes was chosen as follows. The algorithm for computing molecular operational taxonomic units (MOTUs) based on DNA barcode data [49,50] was used to group ~12,600 prokaryotic genomes available in the SEED database in January 2014 into ~1000 such groups. One representative genome from each MOTU was then selected based on the amount of published data (when available) and the level of research interest for different microorganisms within the MOTU. The resulting set of 927 eubacterial and 54 archaeal genomes is a convenient set that accurately represents the diversity of sequenced prokaryotes, and is not skewed by the overabundance of genomes for medically or industrially important organisms (e.g. *E. coli*). The Rid family was analysed in the SEED database within the functional and genomic contexts provided by SEED subsystem ‘RidA family in 981 representative prokaryotes’ available at http://pubseed.theseed.org//SubsysEditor.cgi?page=ShowSubsystem&subsystem=RidA_family_in_981_representative_prokaryotes.

The small size and sequence variability of Rid family proteins preclude accurate detection and annotation of this family in public genome databases. We therefore performed an exhaustive global search for members of this family in all prokaryotic genomes in the SEED database (~8,150 at the time of this work). To do so, 24 diverse representative Rid family DNA sequences were selected (Additional File 3) and used as queries for a global BLAST search of prokaryotic genomes with relaxed search parameters (E-value of e-4) to ensure detection of all relevant targets, including RidA domains in fusions with other proteins. A list of raw BLAST hits was generated (179,483 total) and mapped to the corresponding open reading frames in each of the SEED genomes. The resultant preliminary list of potential RidA-related ORFs (26,879 total) was analyzed and filtered by cross-mapping with the NCBI Conserved Domain Database collection using custom software tools (available upon request) with the goals (i) to filter out false positives (erroneously detected ORFs unrelated to the Rid family); (ii) to classify the Rid family into coherent subgroups ; (iii) to detect fusion events of Rid domains with other protein families; and (iv) to assign accurate consistent annotations to all identified bona fide members of the Rid family in the SEED database (20,658 ORFs total). Based on this analysis, each of the identified Rid family proteins (stand-alone or fused) was assigned to one of eight subfamilies according to the NCBI Conserved Domain Database classification (see cd00448: http://www.ncbi.nlm.nih.gov/Structure/cdd/cddsrv.cgi?uid=100004) and uniformly annotated with one of the Role names from the SEED controlled vocabulary as shown in Table 1.

### Chemicals

Chemicals and enzymes were from Sigma Aldrich (St. Louis, MO).

### cDNAs and expression constructs

For expression of C-terminal His-tagged proteins in *E. coli*, sequences encoding predicted proteins without their stop codons were PCR-amplified (RidA, primers catgccatggctatgagcaaaactattgcgacgg and tccgctcgaggcgacgaacagcgatcgcttcaatc; STM1549, primers catgccatggctatgacgcaxacgtatcgcggtttttcc and tccgctcgagggggattcgggcaataacctttatttcg; YoaB, primers catgccatggctatgtctatcgtgcgtattgatxgc and tccgctcgagtaccgccgcgacaatcttaatctc) from genomic DNA of *S. enterica* subsp. *enterica* serovar Typhimurium str. LT2. Amplicons were digested with *Nco*I and *Xho*I and ligated into the matching sites of pET28b. All constructs were sequence-verified. The ASKA plasmid pCA24N-*carB* was used for CarB overexpression [41]. This plasmid features an IPTG-inducible promoter that controls expression of *carB*, and confers chloramphenicol resistance.

### Bacterial strains

The strains in this study were derivatives of *S. enterica* subsp. *enterica* serovar Typhimurium str. LT2, unless specified otherwise. Complete genotypes are listed in Table 2. MudJ refers to the Mud1734 transposon [51]. Insertion-deletion mutants of *yoaB::cat* and *STM1549::kan* were generated using a previously described method using the pKD3 and pKD4 plasmids, respectively, and the *yoaB::cat* insertion was resolved using the pCP20 plasmid [52]. Mutant strain construction also involved the use of the high frequency general transducing mutant of bacteriophage P22 (HT105/1 int-201) [53], and standard genetic techniques.

**Table 2:** **Bacterial Strains**

**Strain**	**Genotype** ^**a,b**^
**DM14200**	*argI833::tn10 ridA3::MudJ* pCA24N*-carB* (-gfp)
**DM14203**	*argI833::tn10* pCA24N*-carB* (-gfp)
**DM14223**	*argI833::tn10 STM1549-26::kan* pCA24N-*carB* (-gfp)
**DM14307**	*argI833::tn10 ΔyoaB626* pCA24N-*carB* (-gfp)
**DM3480**	*ridA3::MudJ*
**DM14100**	*ridA3::tn10 ΔyoaB624::cat STM1549-26::kan*

^a^MudJ refers to the Mud1734 transposon [1].

^b^pCA24N-*carB* is from the ASKA collection of *Escherichia coli* clones [4].

Strains listed are derivatives of *S. enterica* serovar Typhimurium LT2.

### Protein expression and isolation

Proteins were expressed and purified as previously described [9]. Briefly, *E. coli* strain BL21 (DE3) RIPL harboring each expression construct was grown in 200 mL of LB medium with 50 mg/L kanamycin at 37°C until OD at 600 nm reached 0.8. Cultures were then cooled to 22°C and isopropyl-3-D-thiogalactoside and ethanol were added to final concentrations of 0.5 mM and 4% v/v, respectively. Cultures were incubated for a further 20 h at 22°C and cells were collected and stored at -80°C. Proteins were purified from bacterial lysates with Ni-NTA superflow resin (Qiagen, Valencia, CA) columns according to the manufacturer’s protocol. Proteins were passed through PD-10 columns (GE Healthcare, Cleveland, OH) equilibrated with 5 mM triethanolamine-HCl, pH 7.6, 10% (v/v) glycerol, then concentrated to 80-120 mg/mL with Amicon Ultra-4 3,000 NMWL centrifugal filters (Millipore, Billerica, MA). Aliquots (5 μL) were frozen in liquid nitrogen and stored at -80°C.

### Enzyme assays

To assess the ability of Rid proteins to hydrolyze the imine derived from leucine, asays (100 μL) contained 50 mM potassium pyrophosphate, pH 8.7, 10 mM semicarbazide-HCl (neutralized), 1 μg (3.5 units) bovine liver catalase, 0.5 μg (3 units) *Crotalus adamanteus* LOX, and the indicated amount of *S. enterica* RidA, Rid2, or Rid7. Reactions were started by adding L-leucine (final concentration 5 mM), and absorbance at 248 nm was monitored at 22°C. To assess activity against imines derived from other amino acids, assays were as above except that the amount of LOX was adjusted so that the rate of semicarbazone formation was constant (leucine, 1 μg; methionine, 1.25 μg; phenylalanine, 2 μg; glutamine, 5 μg).

### Growth experiments with *S. enterica*

Cultures were grown at 37°C with shaking in nutrient broth (Difco) containing 20 μg/mL chloramphenicol until stationary phase and then diluted 1:100 in 5 mL of the same medium in 18 x 150 mm borosilicate culture tubes. When present, IPTG was added to a final concentration of 0.1 mM. Growth was monitored by optical density at 650 nm, and analyzed using GraphPad Prism Software (version 6.0).

### Metabolomic analyses

Bacterial cultures were grown overnight in M9 minimal medium plus 0.2% glucose and used to inoculate 2 mL of fresh medium to an optical density of 0.05 at 600 nm. Cultures were grown at 37°C with shaking for 5-6 h until optical density reached 1.7 ± 0.1 at 600 nm, then an equivalent of 1 mL culture at an optical density of 2.0 was collected in 1.5-mL Eppendorf tubes, centrifuged at 16,000 × *g* for 15 s; the pellet was frozen in liquid nitrogen. The harvesting procedure was completed in <30 s. Samples were stored at -80°C. Either six (LC-MS) or 12 (GC-MS) independent cultures for each strain were analyzed. Extraction for LC-MS was conducted by adding 1.1 mL cold, degassed methanol: water (3:1) to each sample and vortexing for 20 sec prior to sonicating for 5 min. Samples were vortexed for another 20 sec then kept at -20°C for 30 min, followed by centrifugation at 14,000 × *g* for 5 min. The resulting supernatant was transferred to 1.5 mL Eppendorf tubes and dried with a LabConco Centrivap Concentrator (LabConco Corporation, Kansas City, MO) overnight. The dried material was dissolved in 100 μL of 5 mM ammonium acetate, 0.2% (v/v) acetic acid and analyzed by LC-MS. Chromatography was performed on an Agilent 1290 Infinity LC System (Agilent Technologies, Santa Clara, CA) using a Waters Acquity 1.7 μm BEH HILIC 150 x 2.1 mm HPLC column as previously described [54]. Extraction for GC-MS analysis was conducted by adding 1 mL of cold, degassed acetonitrile:isopropanol:water (3:3:2) and vortexing for 10 sec. Samples were placed on shaker at 4°C for 4 min then centrifuged at 14000 × *g* for 2 min. Aliquots (450 μL) were transferred to 1.5 mL Eppendorf tubes and dried as above. As previously described [55], the dried material was derivatized with methoxyamine hydrochloride in pyridine followed by *N*-tert-butylmethylsilyl-*N*-methyltrifluoroacetamide and then analyzed by GC-MS. Raw data are available at metabolomicsworkbench.org.

## Additional files

Additional file 1:
**A supplemental table that shows the presence or absence of Rid family genes in 981representative prokaryotic genomes used for the comparative genomics analysis.**
Additional file 2:
**A file that contains material supplemental to this study Figure S1.** Shows purified proteins used in this study. **Table S1.** Lists the relative LOX enzyme activity for various substrates. **Figure S2.** Shows growth of strains used in metabolomics experiments. **Figure S3.** Shows pilot data that suggests Rid proteins cannot hydrolyze carbamoyl phosphate or cyanate. Additional file 3:
**A supplemental table that lists the 24 diverse Rid family sequences used to query bacterial genomes to identify Rid family genes.**
