# Supplementary material for: Genomic and experimental evidence for multiple metabolic functions in the RidA/YjgF/YER057c/UK114 (Rid) protein family
Source: BMC Genomics. 2015 May 15;16(1):382. doi: 10.1186/s12864-015-1584-3 (PMC4433059; doi:10.1186/s12864-015-1584-3)
Supplement: Additional file 2: — A file that contains material supplemental to this study Figure S1. Shows purified proteins used in this study. Table S1. Lists the relative LOX enzyme activity for various substrates. Figure S2. Shows growth of strains used in metabolomics experiments. Figure S3. Shows pilot data that suggests Rid proteins cannot hydrolyze carbamoyl phosphate or cyanate. [file 12864_2015_1584_MOESM2_ESM.pdf]

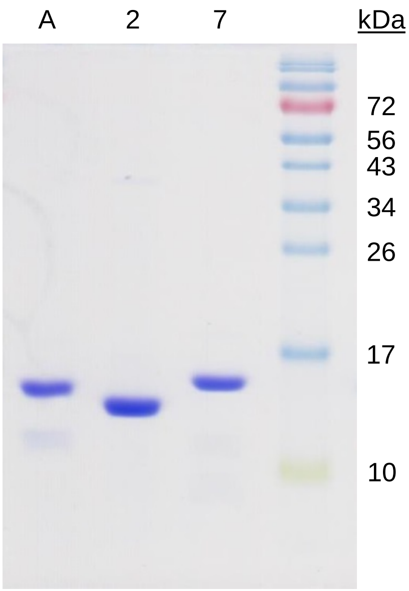

Supplemental Figure 1. Recombinant proteins used in this study were purified to near homogeneity. 5 µg of Ni-affinity purified *Salmonella enterica* RidA (A), Rid2 (2), and Rid7 (7) were analyzed by SDS-PAGE (15% gel) with Coomassie staining. Each protein preparation was judged to be ≥90% pure.

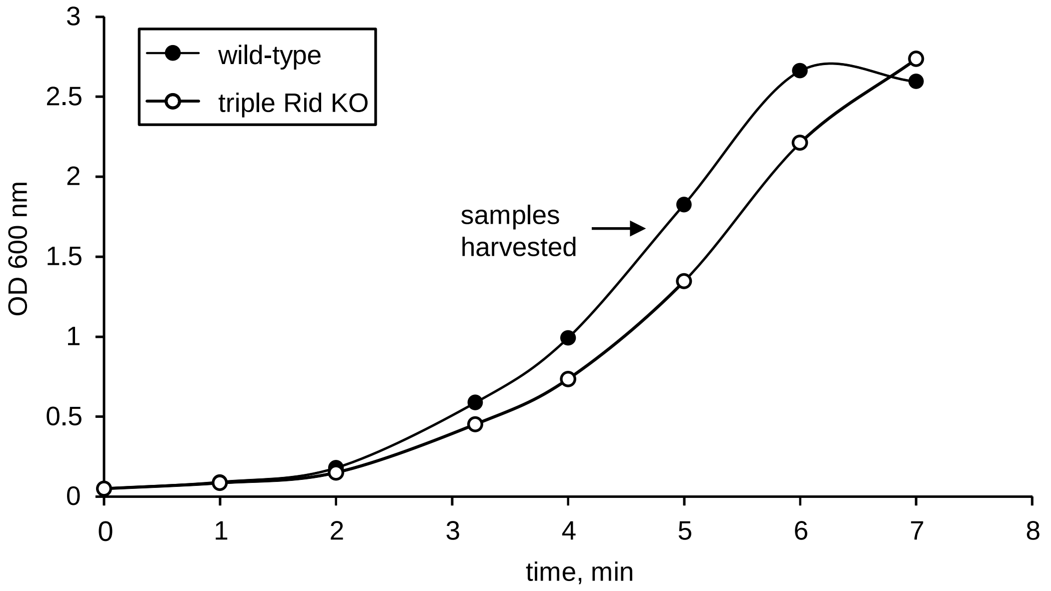

Supplemental Figure 2. Growth of wild-type *S. enterica* and triple Rid KO (*ridA Rid2 Rid7*; DM14100) cells in M9 medium (0.2% glucose). Fresh medium was innoculated with overnight cultures to an OD (600 nm) of 0.05 and cultures were grown at 37°C. Data shown are representative of at least three independent experiments done on separate days. For metabolomics, samples were harvested at OD 600 nm = 1.7±0.1.

A

Rate of cyanate breakdown in presence of Rid proteins	
sample	µmol NH_3 formed
control	<1.0
RidA	<1.0
Rid2	<1.0
Rid7	<1.0

B

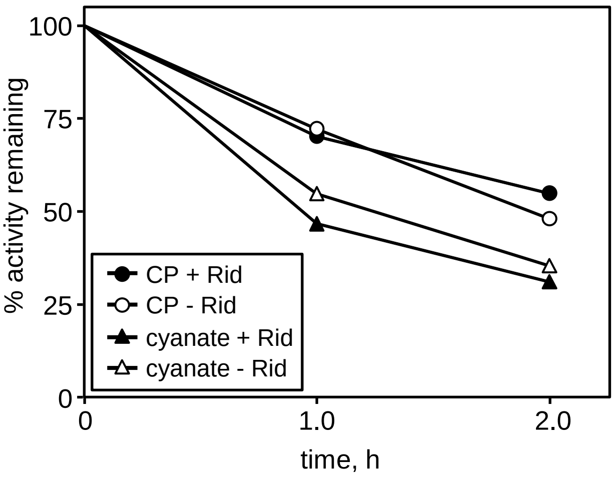

Supplemental Figure 3. *Salmonella enterica* Rid proteins do not increase the rate of breakdown of cyanate or carbamoyl phosphate. **(A)** Assays (50 µL) contained 50 mM KPi, pH 8.0, 100 µM sodium cyanate, with or without 100 µM *S. enterica* RidA, Rid2, or Rid7 and were incubated for 30 min at 22°C. Afterwards, 50 µL of GDH master mix containing 50 mM Kpi, pH 8.0, 7 mM 2-oxoglutarate, 200 µM NADPH, and 6 U L-glutamic dehydrogenase from bovine liver (Sigma-Aldrich) was added and the optical density at 340 nm was monitored for 5 min at 22°C. The amount of NADPH oxidized was calculated (ε340 nm = $6200 \cdot M^{-1} \cdot cm^{-1}$). **(B)** Preincubation reactions (25 µL) contained 50 mM KPi, pH 7.5, 1 mM isoleucine, 10 µM *Arabidopsis thaliana* threonine dehydratase [9], with (closed symbols) or without (open symbols) 100 µM each of *S. enterica* RidA, Rid2, and Rid7, and 25 mM of either carbamoyl phosphate (CP, circles) or cyanate (triangles) and were incubated at 37°C. At the indicted times, 5 µL of preincubation assay was added to 95 µL of assay mixture containing 50 mM KPi, pH 8.0, 5 mM threonine, and the absorbance at 248 nm was recorded at 22°C. Data is reported as the percent of the rate of 2-oxobutanoate formed at t=0.

Table S1

Relative rate of semicarbazone formation for various amino acid substrates incubated with *Crotalus adamanteus* L-amino acid oxidase.

**Amino acid**	**Relative rate** [a]
Leu	100
Met	79.1
Phe	46.1
Lys	6.5
Ser	0.4
Glu	0.3
Gln	21.3

[a]rate is expressed as a percent of the rate of semicarbazone formation with Leu as the substrate
